# Toxic effects of atrazine on porcine oocytes and possible mechanisms of action

**DOI:** 10.1371/journal.pone.0179861

**Published:** 2017-06-22

**Authors:** Bao Yuan, Shuang Liang, Yong-Xun Jin, Ming-Jun Zhang, Jia-Bao Zhang, Nam-Hyung Kim

**Affiliations:** 1 Department of Laboratory Animals, College of Animal Sciences, Jilin University, Changchun, Jilin, China; 2 Molecular Embryology Laboratory, Department of Animal Sciences, Chungbuk National University, Cheongju, Chungbuk, South Korea; Institute of Zoology Chinese Academy of Sciences, CHINA

## Abstract

Because atrazine is a widely used herbicide, its adverse effects on the reproductive system have been extensively researched. In this study, we investigated the effects of atrazine exposure on porcine oocyte maturation and the possible mechanisms. Our results showed that the rates of oocyte maturation significantly decreased after treatment with 200 μM atrazine in vitro. Atrazine treatment resulted in abnormal spindle morphology but did not affect actin distribution. Atrazine exposure not only triggered a DNA damage response but also decreased MPF levels in porcine oocytes. Our results also revealed that atrazine worsened porcine oocyte quality by causing excessive accumulation of superoxide radicals, increasing cathepsin B activity, and decreasing the GSH level and mitochondrial membrane potential. Furthermore, atrazine decreased developmental competence of porcine oocytes up to the blastocyst stage and changed some properties: cell numbers, apoptosis, and related gene expression levels. Collectively, our results indicate that porcine oocyte maturation is defective after atrazine treatment at least through disruption of spindle morphology, MPF activity, and mitochondrial function and via induction of DNA damage, which probably reduces developmental competence.

## Introduction

Atrazine (2-chloro-4-ethylamino-6-isopropyl-amino-s-triazine) is a cheap herbicide that is widely used worldwide to control crop weeds in the field, but because of its possible harmful effects, it is currently banned in some countries [1–3]. Atrazine is thought to have only mild toxicity, although several studies have shown that it may damage an animal’s reproductive system [4]. Atrazine may affect the frog gonadal development [5] by inducing aromatase-driven development, and this process increases the conversion of androgen to estrogen [6, 7]. Because many studies have shown that atrazine can affect animal endocrine function, especially in the thyroid and reproductive systems, atrazine has also been shown to interfere with the functioning of the endocrine system [8–11]. The effect of atrazine on the reproductive system is mainly mediated by the influence on steroid synthesis [12].

Atrazine is also capable of competitive inhibition of cyclic nucleotide phosphodiesterase [13–15]. In rat testicular Leydig cells or in the pituitary gland, atrazine increases the production of prolactin and testosterone by inhibiting cAMP-specific phosphodiesterase 4, thereby increasing the cAMP stores in the cell [16, 17]. The increase in cAMP and androgen production in Leydig cells was also observed after short in vivo treatment with atrazine [18, 19]. Although in vivo toxicity studies can better reflect the toxic effects of pesticides on experimental animals or field workers, the use of an in vitro system of pig oocyte maturation may be a simpler way to identify pesticides toxic for the reproductive system [20–22].

A few studies have shown toxic effects of atrazine on oocyte quality in vitro in *Xenopus* frogs and Japanese medaka models [23–25]. These studies have revealed that atrazine is detrimental to oocyte maturation; however, the mechanisms of toxicity are still not clear. To determine the mechanism underlying the toxic effects of atrazine on oocytes, adverse effects on both meiotic characteristics and cytoplasmic components should be examined. Because the porcine genome is rather similar to the human genome, such experiments could more accurately reflect the reproductive system of humans than experiments on rodents can.

Therefore, the aim of this study was to assess the influence of atrazine on porcine oocyte maturation; these data could expand our knowledge about the mechanisms of atrazine’s action on the reproductive system.

## Materials and methods

### Ethics statement

This study’s protocol was approved by the IACUC of Jilin University (Permit Number: 20160406).

### Reagents

Unless otherwise stated, all the reagents were purchased from Sigma-Aldrich (St. Louis, MO, USA).

### Collection and in vitro maturation (IVM) of porcine oocytes

The ovaries of the pigs were obtained from a slaughterhouse, stored in physiological saline supplemented with 1% of an antibiotic solution, and sent to the laboratory within 3 h. Porcine cumulus-oocyte complexes (COCs) were recovered from porcine follicles with a diameter of 3–6 mm and were washed three times with Tyrode’s Lactate HEPES (TL-HEPES) supplemented with gentamycin (0.05 g/L) and polyvinyl alcohol (PVA, 1 g/L); each time, precipitation lasted for 10 min. The collected COCs were maturated in the IVM medium for 44 h at 38.5°C in a humidified atmosphere of 5% CO_2_/95% air. The IVM medium was based on the modified TCM-199 medium [16] and contained 108.73 mM NaCl, 25.07 mM NaHCO_3_, 4.78 mM KCl, 1.19 mM KH_2_PO_4_, 1.19 mM MgSO_4_, 1.70 mM CaCl_2_, and 1.00 mM glutamine. This medium was stored at 4°C and used within 2 weeks of preparation. Before use, the medium was supplemented with 0.6 mM L-cysteine, 10 ng/mL epidermal growth factor, 10 IU/mL luteinizing hormone, 10 IU/mL follicle-stimulating hormone, 5 mg/mL insulin, and 10% v/v porcine follicular fluid.

According to other studies, various concentrations of atrazine (0, 50, 100, 200, or 500 μM) were added to the IVM medium. After 42–44 h of IVM, the COCs were washed with TL-HEPES supplemented with 1 mg/mL hyaluronidase and 0.1% of PVA to remove cumulus cells. Oocytes that had discharged the first polar body were selected for further experiments.

### Parthenogenetic activation and in vitro culture of pig oocytes

In accordance with the activation protocol used in our previous studies, meiosis II (MII) stage oocytes were used [26]. Denuded oocytes with homogeneous cytoplasm were selected, gradually equilibrated in the activation solution, and subjected to a 1.2 kV/cm electric pulse for 60 μs. The activated oocytes were incubated in the PZM-5 medium with 7.5 μg/ml cytochalasin B for 3 h. Next, 40–50 postactivation oocytes were cultured in PZM-5 for another 7 days, and the resultant embryo cultures were maintained at 38.5°C and 5% CO_2_.

### Immunofluorescent staining

The oocytes were washed with phosphate-buffered saline (PBS) and fixed in 3.7% paraformaldehyde (w/v) prepared in PBS containing 0.1% PVA and treated with 1% Triton X-100 (v/v) at 37°C for 1 h. The samples were blocked with 1% BSA (w/v) for 1 h and incubated overnight at 4°C with different antibodies in a blocking solution. For spindle and actin examination, the oocytes were incubated with a FITC-conjugated anti-α-tubulin antibody and phalloidin-TRITC. For DNA damage examination, the oocytes were incubated with an anti-γH2AX antibody (pS139, 1:100; Cell Signaling Technology), then washed three times with PBS containing 1% BSA, and incubated with an FITC-conjugated secondary antibody (1:100) at room temperature for 1 h. Next, the samples were counterstained with Hoechst 33342 (10 μg/ml in PBS) for 15 min, followed by three washes with PVA–PBS. After that, the oocyte samples were mounted on a glass slide, and examined under an LSM 710 META confocal laser-scanning microscope (Zeiss, Jena, Germany).

### Quantification of reactive oxygen species (ROS), GSH, cathepsin B activity, and membrane potential in MII oocytes

To determine the influence of atrazine on metabolic pathways, MII stage oocytes were transferred to a medium supplemented with different reaction mixtures. Briefly, the oocytes were incubated in 500 μL of PBS-PVA supplemented with 2 μL of the reaction mixture in a humidified atmosphere containing 5% CO_2_ at 39°C for 20 min. To detect the levels of ROS, the oocytes were incubated with 10 μM 2′,7′-dichlorodihydrofluorescein diacetate (H2DCFDA; Thermo Fisher Scientific, Waltham, USA) for 15 min, followed by spectroscopy (green fluorescence, UV filters, 490 nm). To measure the levels of GSH, the oocytes were incubated with 10 μM 4-chloromethyl-6,8-difluoro-7-hydroxycoumarin (CMF_2_HC) Cell Tracker Blue dye (Thermo Fisher Scientific) for 15 min, followed by spectroscopy (blue fluorescence, UV filters, 370 nm). The cathepsin B activity in oocytes was quantified using a commercial Magic Red Cathepsin B Assay Kit (ImmunoChemistry Technologies LLC, Bloomington, MN, USA). To measure mitochondrial membrane potential (Δφm), the oocytes were incubated with 1 mM/L 5,5′,6,6′-tetrachloro-1,1′,3,3′-tetraethyl-imidacarbocyanine iodide (JC-1; Invitrogen). Δφm was calculated as the ratio of red fluorescence corresponding to activated mitochondria (J-aggregates) to green fluorescence corresponding to the less activated mitochondria (J-monomers). After incubation, the oocytes were washed three times in PBS-PVA. The fluorescence intensity of oocytes was analyzed in the ImageJ software (public domain). The oocytes of the different groups were processed identically with respect to incubation, washing, mounting, and imaging. Three independent experiments were conducted.

### Assay of maturation-promoting factor (MPF) activity in MII oocytes

We used the Cdc2/Cdk1 Kinase Assay Kit (MBL, Nagoya, Japan) to quantify p34^cdc2^ kinase activity [27, 28]. Briefly, 30 oocytes were washed three times with sample buffer. The oocyte extract (5 μL) was mixed with kinase assay buffer (45 μL). The mixture was placed in an incubator at 30°C and kept there for 30 min. The reaction was terminated by the addition of 200 μL of 50 mM ethylene glycol tetraacetic acid (EGTA). Optical density (OD) was measured at 492 nm. Three independent experiments were conducted.

### Real-time reverse transcription PCR

Total RNA extraction and cDNA synthesis were performed as described previously [29]. Briefly, mRNA was extracted from 50 MII oocytes or 20 blastocysts by means of the Ambion Dynabeads mRNA Direct Kit (Thermo Fisher Scientific). The isolated mRNA was reverse-transcribed with the SuperScript Reverse Transcriptase Kit (LeGene Biosciences, San Diego, CA, USA), using oligo(dT)_12–18_ primers, followed by real-time PCR on a Bio-Rad CFX PCR cycler (Hercules, CA, USA) with the primers that were described in our previous paper [26]. Gene expression was analyzed by the 2^−ΔΔCt^ method [30], using data from the glyceraldehyde 3-phosphate dehydrogenase (*GAPDH*) gene for normalization. Three separate experiments were carried out, each containing three samples.

### Confocal-microscopy quantification of nuclei per blastocyst

We performed these procedures as described elsewhere [26]. Briefly, blastocysts were fixed for 30 min in 3.7% paraformaldehyde prepared in PBS with 0.1% (w/v) PVA (PBS-PVA). After that, they were washed with PBS, and then incubated with 0.3% Triton X-100 for 1 h. Next, they were washed twice with PBS and incubated in the dark for 1 h after the addition of fluorescein-conjugated dUTP and terminal deoxynucleotidyl transferase (a TUNEL assay). After a 1-h incubation with 10 μg/mL Hoechst 33342 and 50 mg/mL RNase A, the blastocysts were examined under a laser scanning confocal microscope. The total number of cells and the number of apoptotic cells were determined using the ImageJ software.

### 5-Bromo-deoxyuridine (BrdU) assay

This assay was conducted as previously described [31] with minor modifications. Briefly, blastocysts were incubated in 100 mM BrdU at 5% CO_2_ and 39°C for 6 h. The blastocysts were washed with PBS containing 0.1% Tween 20 (PBST), fixed in ice-cold methanol for 20 min, washed again, and then permeabilized at room temperature (20–25°C) for 2 min with 0.1% Triton X-100 (prepared in PBS-PVA). The blastocysts were then washed and treated with 2N HCl at room temperature for 30 min. After a wash, they were incubated with a mouse anti-BrdU monoclonal antibody at a dilution of 1:10 overnight at 4°C. Incubation with the secondary antibody (a G-FITC-conjugated rabbit anti-mouse immunoglobulin polyclonal antibody, 1:500 dilution) was performed at room temperature for 1 h. Finally, the blastocysts were stained with 10 μg/mL Hoechst 33342, fixed on glass slides, and examined under a confocal laser scanning microscope. The number of proliferating cells was determined using ImageJ.

### Statistical analyses

All the experimental results were analyzed in IBM SPSS Statistics v.19 (IBM, Armonk, NY, USA). One-way ANOVA and Chi-square test were applied to evaluate the statistical significance of differences; all percentage data were subjected to arc sine transformation before statistical analysis. A difference with a p value lower than 0.05 was considered statistically significant.

## Results

### Effects of atrazine on IVM of porcine oocytes

**Fig 1:**
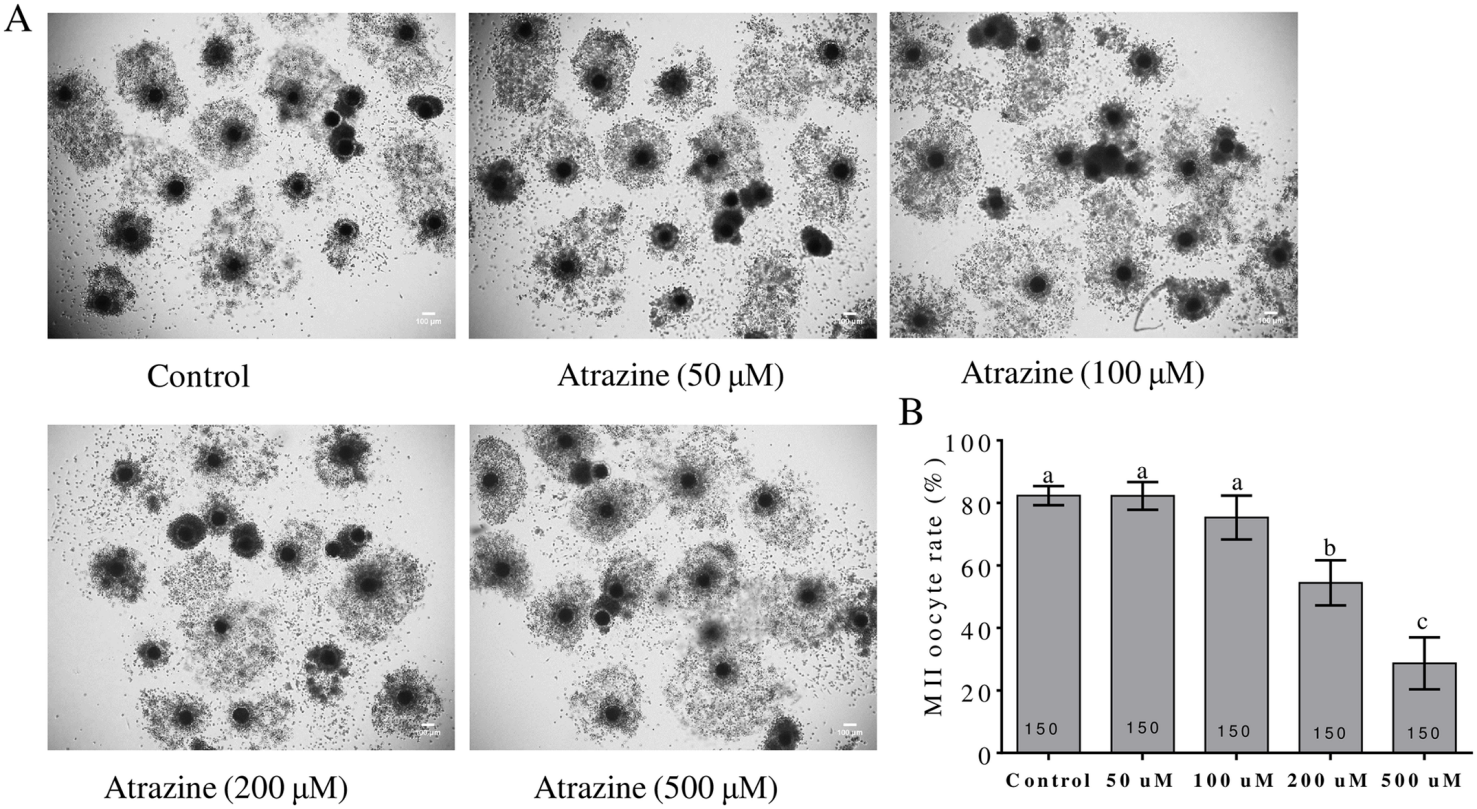
Effects of various concentrations of atrazine on IVM of porcine oocytes. (A) Expansion of the peripheral layers of cumulus cells. (B) The polar body extrusion rate was significantly reduced after atrazine treatment. Control: a normal IVM medium; 50–500 μM: different concentrations of atrazine. Differences between bars superscripted with different letters (a, b, or c within the same graph) are statistically significant (p < 0.05). Values are shown as mean ± standard deviation from three independent experiments. The number of oocytes observed in each experimental group is displayed in the bar.

To evaluate this effect, COCs were maturated in the presence of different concentrations of atrazine (0, 50, 100, 200, or 500 μM). As shown in Fig 1A, good expansion of the peripheral layers of cumulus cells was observed in each maturation group. Even after treatment with 500 μM atrazine, there was no effect on expansion of the peripheral layers of cumulus cells. Nonetheless, most of oocytes had extruded polar bodies and were arrested at the MII stage in control, 50-μM, and 100-μM atrazine groups, whereas in the 200-μM atrazine group, polar body extrusion was suppressed in the oocytes (Fig 1B). The 200 μM atrazine-treated group showed a significant decrease in the rates of nuclear maturation (54.00 ± 7.21% vs. 81.33 ± 3.06%, p < 0.05). Therefore, for the subsequent experiments, we selected 200 μM atrazine as the treatment dose.

### Effects of atrazine on the spindle and actin of MI stage oocytes

**Fig 2:**
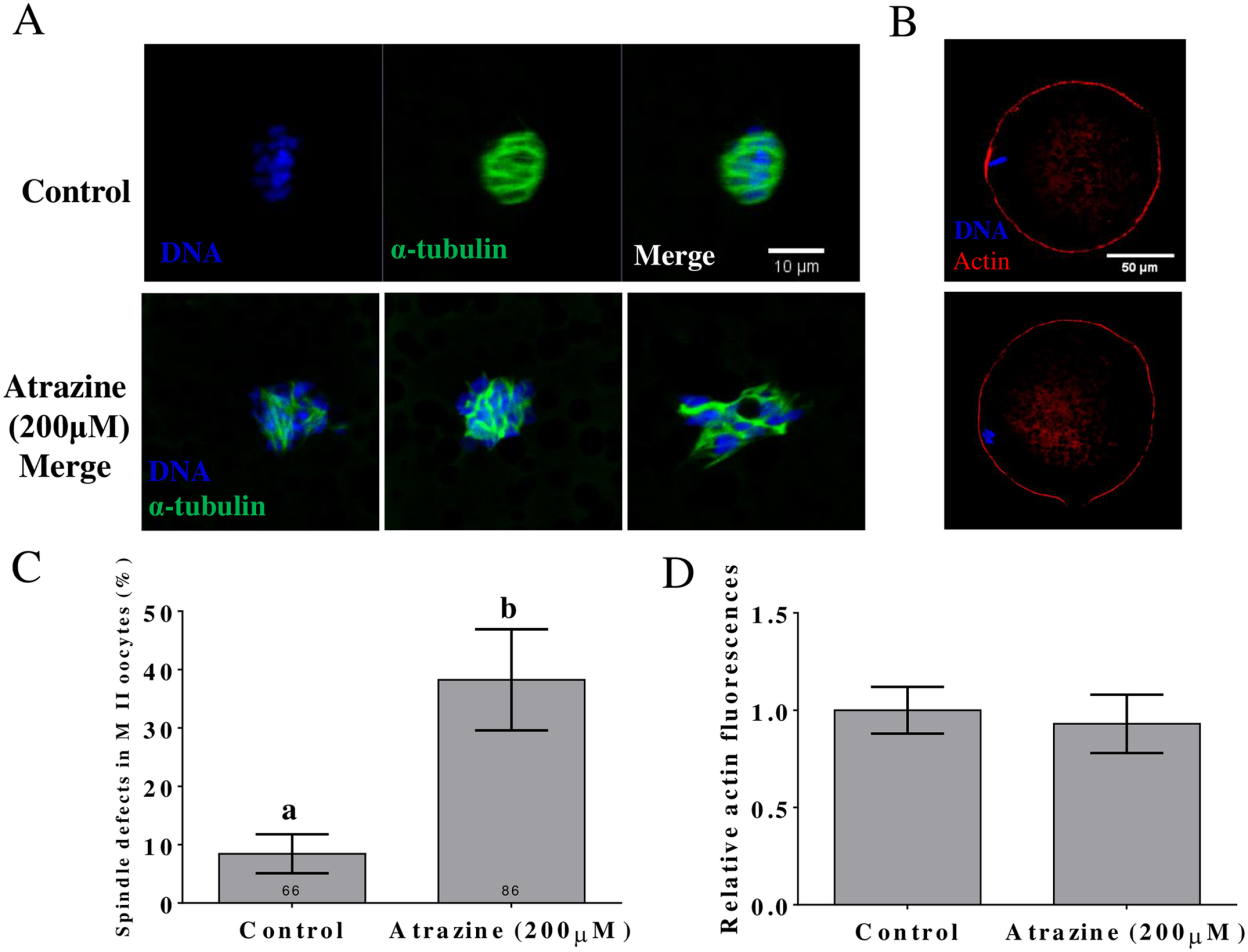
Atrazine causes oocyte spindle abnormalities during porcine oocyte maturation. (A) Spindle formation after atrazine treatment. (B) Actin signals after atrazine treatment. (C) Rate of abnormal spindle formation was significantly increased. (D) Relative actin fluorescence signals in the membrane and in the cytoplasm of meiosis I (MI) oocytes. Differences between bars superscripted with different letters (a or b within the same graph) are statistically significant (p < 0.05). The number of oocytes observed in each experimental group is displayed in the bar. Values are presented as mean ± standard deviation from three independent experiments. Blue, chromatin DNA; green, tubulin; red, actin.

We explored the influence of melatonin supplementation on porcine oocyte cytoskeletal integrity after IVM. In porcine oocytes, we first assessed meiotic spindle organization. Fig 2A shows the results of the analysis of spindle morphology. A large proportion of oocytes had spindle abnormalities after treatment with 200 μM atrazine. In the 200 μM atrazine-treated group, the proportion of oocytes with spindle abnormalities was dramatically higher than that in the untreated group at 28 h (p < 0.05; Fig 2C). Nevertheless, the relative actin fluorescence was not changed significantly (p > 0.05; Fig 2B and 2D).

### Damaging effects of atrazine on DNA of oocytes at the germinal-vesicle breakdown (GVBD) stage

**Fig 3:**
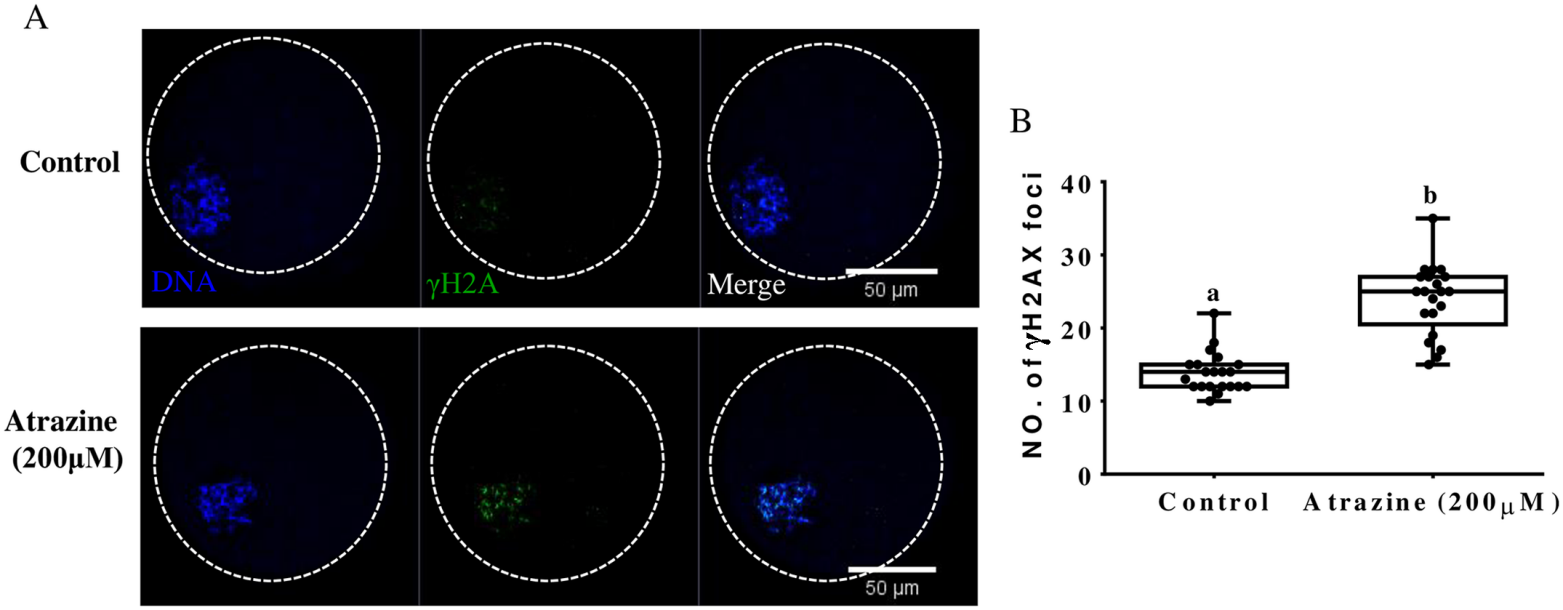
Damaging effects of atrazine on DNA at the GVBD stage. (A) DNA damage levels (as indicated by H2A.X fluorescence intensity). (B) Number of γH2AX foci in different groups. Differences between bars superscripted with different letters (a or b within the same graph) are statistically significant (p < 0.05). Values are presented as mean ± standard deviation from three independent experiments. Blue, chromatin DNA; green, H2A.X signals.

To study the relation between oocyte quality and atrazine exposure, in addition, we examined the changes in DNA damage (as indicated by γH2A.X) at the GVBD stage. Supplementation with 200 μM atrazine significantly increased DNA damage (p < 0.05; Fig 3A and 3B) at 20 h as compared with that in untreated oocytes.

### Effects of atrazine on maternal gene expression and MPF activity

**Fig 4:**
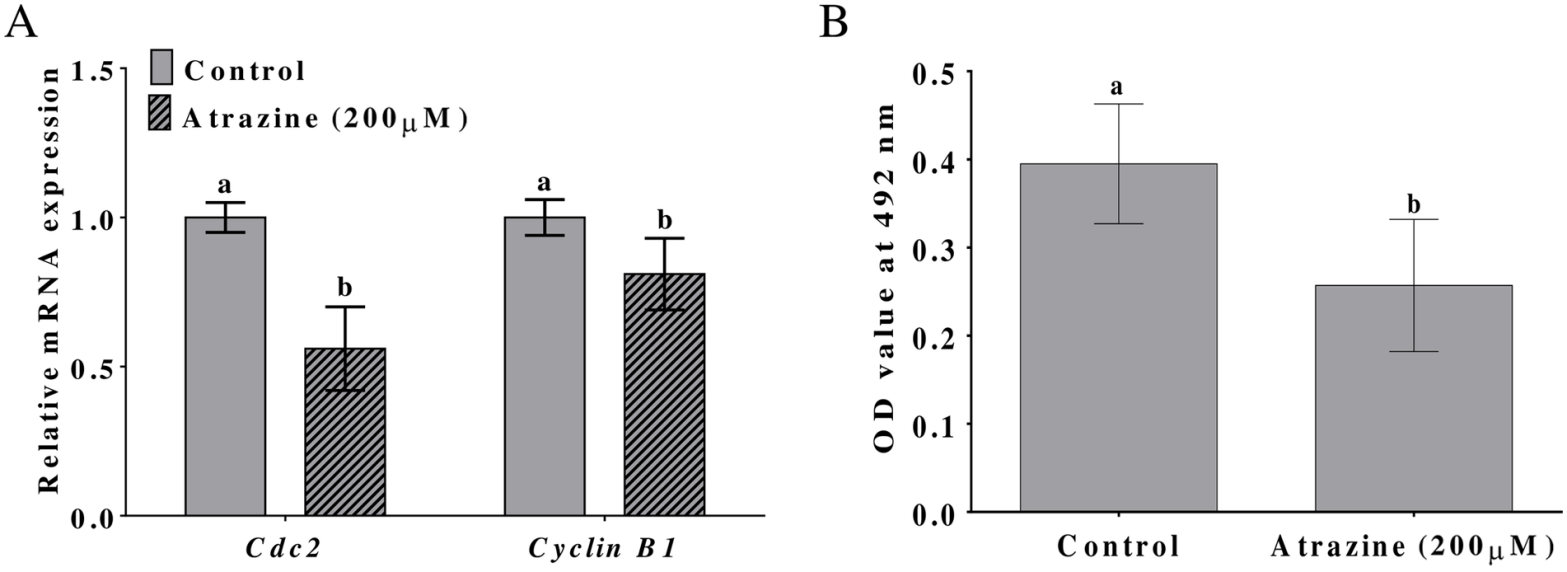
Effects of atrazine on maternal gene expression and MPF activity. (A) Expression levels of the maternal transcripts of cdc2 and cyclin B1. (B) MPF activity in MII oocytes, after maturation of porcine COCs in the presence of 200 μM atrazine. Differences between bars superscripted with different letters (a or b within the same graph) are statistically significant (p < 0.05). Values are presented as mean ± standard deviation from three independent experiments.

The regulation of maternal gene expression is an important biological process during oocyte maturation and early embryonic development. We examined the effects of 200 μM atrazine on this process by evaluating the expression of two representative maternal transcripts, cdc2 and cyclin b1, which encode the regulatory subunits of MPF. The mRNA levels were measured in MII oocytes after IVM of COCs in the presence of 200 μM atrazine. As shown in Fig 4A, 200 μM atrazine significantly (p < 0.05) reduced cdc2 and cyclin b1 mRNA levels as compared to that in no-treatment controls. We also measured the activity of p34cdc2a, which is the protein encoded by the *cdc2* gene. MII oocytes that had been treated with 200 μM atrazine during IVM displayed significantly reduced (p < 0.05) p34cdc2a activity as compared with that in the control group (Fig 4B).

### Effects of atrazine on intracellular levels of ROS, GSH, and cathepsin B

**Fig 5:**
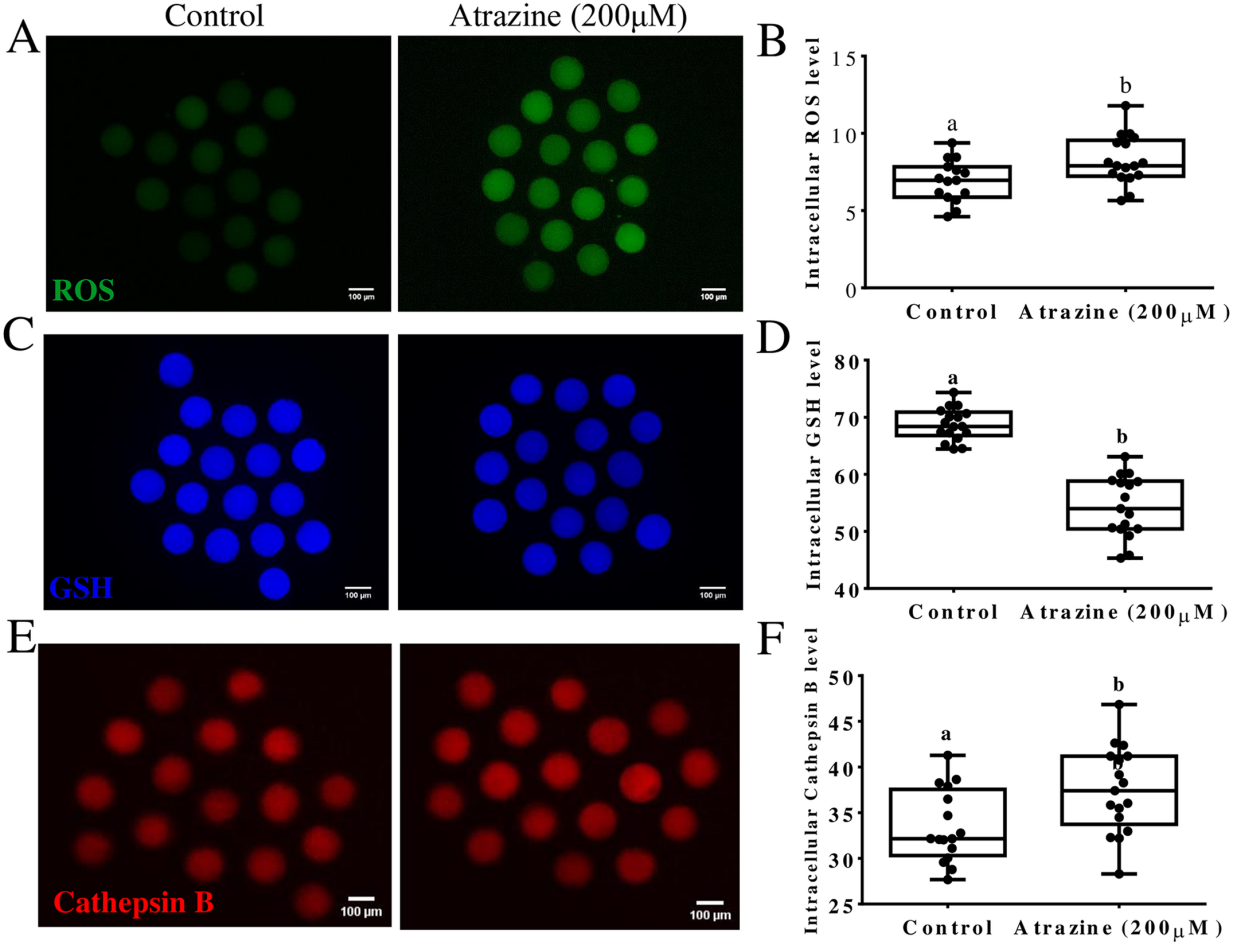
Effects of atrazine on intracellular levels of ROS, GSH and cathepsin B activity. (A) MII oocytes maturated in the normal IVM medium or the medium supplemented with 200 μM atrazine were stained with H2DCFDA to evaluate ROS levels. (B) Effects of 200 μM atrazine supplementation during IVM on intracellular ROS levels in mature oocytes. (C) MII oocytes were stained with CMF2HC (Cell Tracker Blue) to assess GSH levels. (D) GSH levels in mature oocytes. (E) MII oocytes were stained with Magic red cathepsin B. (F) Cathepsin B levels in mature oocytes. Differences between bars superscripted with different letters (a or b within same graph) are statistically significant (p <0.05). The experiment was repeated three times.

To determine the mechanism of 200 μM atrazine’s action on porcine oocyte maturation, concentrations of GSH, ROS, and cathepsin B were determined in the corresponding post-IVM oocytes. The levels of ROS were significantly higher (p < 0.05) in the 200 μM atrazine-treated oocytes (8.26 ± 0.38 pixels per oocyte) than in the control group (6.90 ± 0.34 pixels per oocyte; Fig 5A and 5B). In contrast, GSH was significantly downregulated (p < 0.05) in 200 μM atrazine-treated group (54.34 ± 1.29 pixels per oocyte) than in the control group (68.73 ± 0.68 pixels per oocyte; Fig 5C and 5D). The amounts of cathepsin B were significantly (p < 0.05) higher in the 200 μM atrazine-treated oocytes (37.49 ± 1.14 pixels per oocyte) than in the control group (33.47 ± 0.99 pixels per oocyte; Fig 5E and 5F).

### Influence of atrazine on Δφm

**Fig 6:**
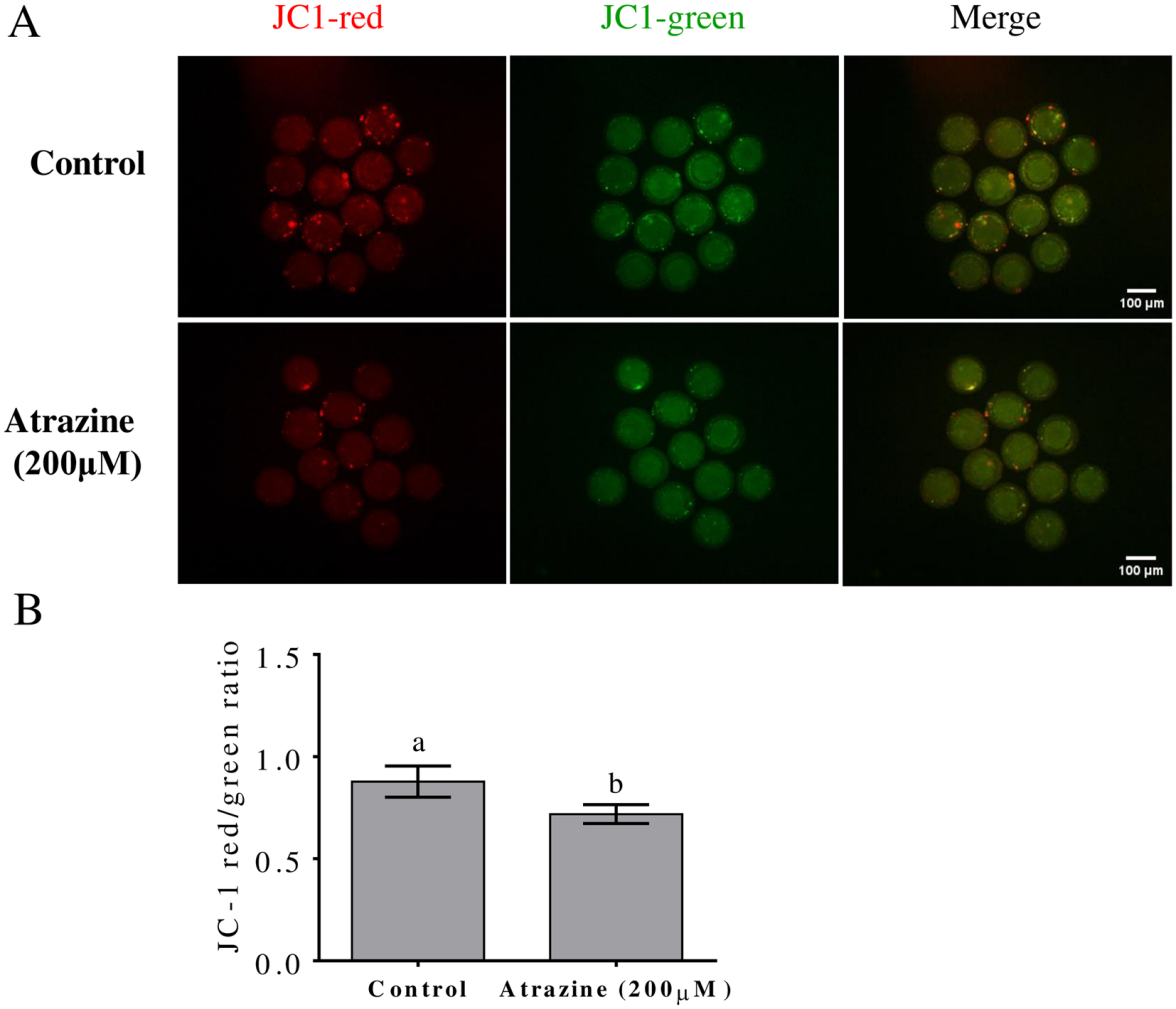
Effects of atrazine on mitochondrial membrane potential (Δφm). (A) The membrane potential was calculated as the ratio of red fluorescence, green fluorescence, and merged data. (B) Fluorescence emitted by each MII oocyte was analyzed by means of the ImageJ software (red/green). Differences between bars superscripted with different letters (a or b within same graph) are statistically significant (p < 0.05). The experiment was repeated three times.

To elucidate the mechanism behind atrazine’s effect on porcine oocyte maturation, Δφm values in MII oocytes were measured. Δφm was determined in 200 μM atrazine-treated oocytes by JC-1 staining (Fig 6A). Compared with that in the control, 200 μM atrazine significantly decreased (p < 0.05) Δφm (Fig 6B).

### Effects of atrazine administered during IVM on subsequent early embryonic development

**Fig 7:**
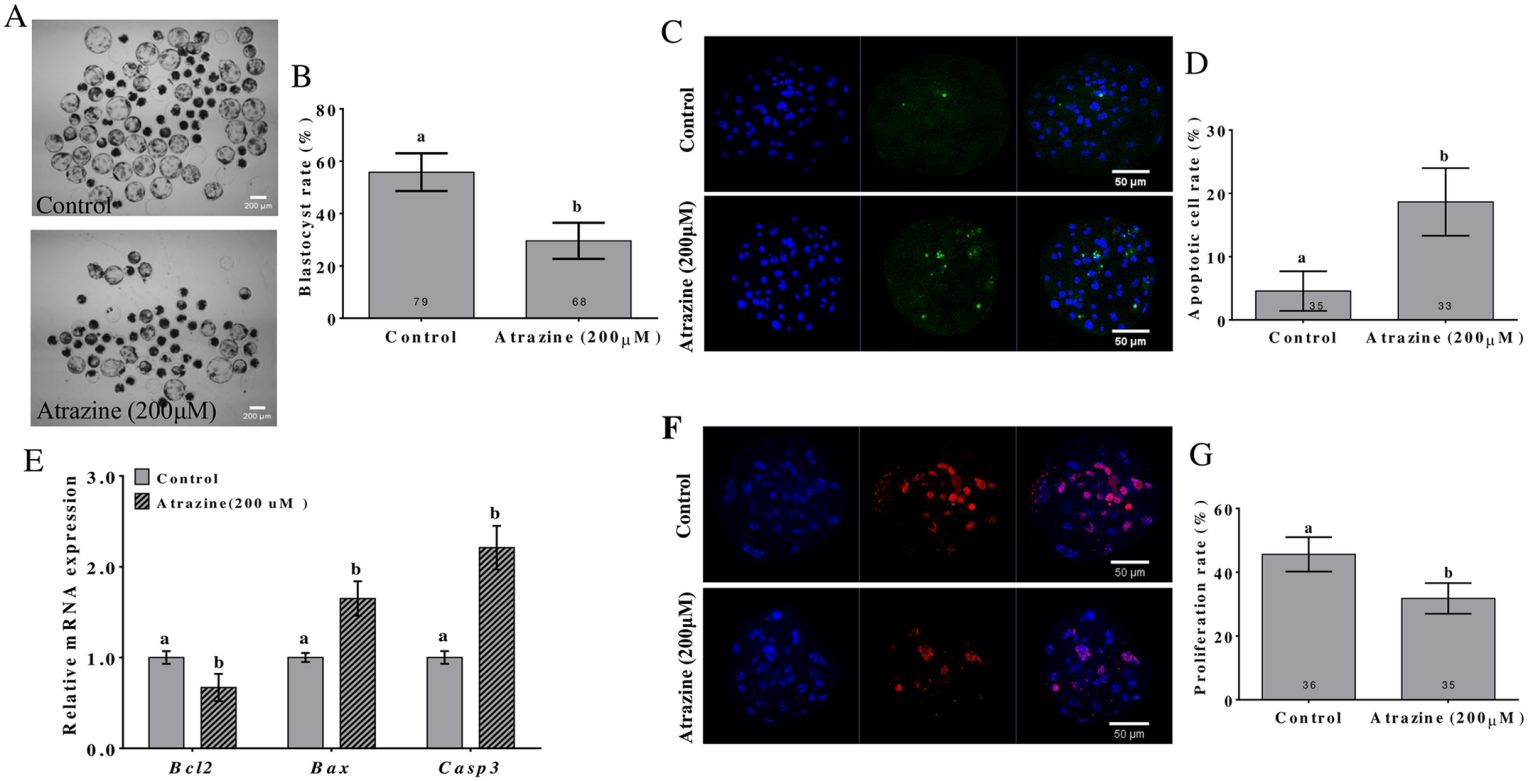
Effects of atrazine administered during IVM on subsequent early embryonic development. (A) Blastocysts when COCs were cultivated in the normal IVM medium or medium supplemented with 200 μM atrazine. (B) The blastocyst rate in two groups. (C) Immunofluorescent staining of apoptotic cells in blastocysts from control and 200 μM atrazine-treated groups (×400). (D) Apoptotic cell rates per blastocyst in different groups. (E) *Bcl-2*, *Bax*, and *Casp3* mRNA amounts in 7-day porcine blastocysts in controls and groups treated with 200 μM atrazine during IVM. (F) Immunofluorescent staining of BrdU in blastocysts from control and 200 μM atrazine-treated groups (×400). (G) Percentages of proliferating cells in different groups. Values are presented as mean ± standard deviation. The numbers of embryos examined in each experimental group are shown in the bars. Differences between bars superscripted with different letters (a or b) are statistically significant (p < 0.05).

To determine whether 200 μM atrazine treatment during IVM affected subsequent embryonic development, porcine oocytes were activated, and their in vitro development was examined (Fig 7). In the IVM medium without 200 μM atrazine, the blastocyst rate was significantly higher than that in the 200 μM atrazine-treated group (55.82% ± 7.20% vs. 29.58% ± 6.90%, p < 0.05; Fig 7A and 7B). The DNA fragments produced by the apoptotic incision of genomic DNA were quantified in a single embryo by the TUNEL assay. Compared with the control group, the 200 μM atrazine treatment group showed a significantly higher (p < 0.05) apoptotic cell rate (Fig 7C and 7D). To identify the mechanism of atrazine’s proapoptotic action on the parthenogenetic blastocysts, we measured the expression levels of apoptosis-related genes *Bcl2*, *Bax*, and *Casp3*. Compared with that in the untreated group, the *Casp3* and *Bax* mRNA levels were significantly higher in the 200 μM atrazine-treated group, and the *Bcl-2* mRNA amount was significantly lower (p < 0.05; Fig 7E). We evaluated the cell proliferative capacity by the BrdU assay. Representative images of proliferation of blastocysts are shown in Fig 7F. In the 200 μM atrazine-treated group, the percentage of proliferating cells was significantly lower than that in the untreated group (p < 0.05; Fig 7G).

## Discussion

Atrazine, a weed inhibitor (a triazine herbicide) acting on light system II, has been used for nearly 60 years, and is probably the world’s most commonly used herbicide [32, 33]. Atrazine is an endocrine disruptor that can impair human and animal reproductive capacity [12, 18, 34, 35]. In the present study, our results indicated that exposure to atrazine caused death of porcine oocytes by inhibiting polar body extrusion. Subsequently, the toxicity of atrazine was demonstrated and its potential mechanisms were explored, including the cytoskeleton, maternal gene expression levels, oxidative stress, Δφm, and further embryonic properties by determining developmental rates, cell numbers, apoptosis, and the related gene expression.

Although our results showed that the expansion of cumulus cells was not affected, polar body extrusion was impaired in porcine oocytes after atrazine treatment. Our results indicate that just as in other models, in our experimental model, atrazine has direct toxic effects on porcine oocytes in vitro [23, 25]. Similar results have been reported in porcine oocytes, namely, that treatment with atrazine decreases oocyte meiotic maturation [36]. In mice, oocytes exposed to high doses of atrazine undergo a cell cycle delay and manage to progress to MII when the incubation period is prolonged [37, 38]. Therefore, our study and other studies show that a high concentration of atrazine blocks the cell cycle progression during porcine oocyte maturation. This finding indicates that porcine oocytes are sensitive to the atrazine exposure.

We found that high concentrations of atrazine significantly increased the frequency of γ-H2AX foci, suggesting that atrazine can disrupt DNA integrity in oocytes and may be cytotoxic. Disrupted DNA integrity can result in chromatin remodeling, cell cycle delay, or cell death [39–42]. Some reports suggest that double-strand breaks (DSBs) in DNA are related to ROS generated after exposure to pesticides [43–45]. The primary endogenous source of ROS is mitochondria; therefore, we tested whether atrazine affected the functioning of these organelles. Δφm provides the basis for mitochondrial respiration, which allows ADP to be converted to ATP by respiratory enzymes. Δφm depolarization disrupts electron transfer to receptor oxygen molecules, resulting in excessive ROS production. In this study on porcine oocytes, atrazine treatment caused greater depolarization of mitochondria and increased ROS abundance. These data suggest that atrazine adversely affects electron transfer within mitochondria; this phenomenon can explain excess ROS production.

MPF activity performs an important function in the cell cycle. In this study, we found that treatment with atrazine significantly decreased mRNA levels of the maternal gene *cdc2* as well as the activity of the p34^cdc2^ kinase (which is encoded by *cdc2*). These results suggest that atrazine’s detrimental effects may affect the maturation of porcine oocytes by regulating MPF activity.

Our study indicates that exposure to atrazine during oocyte maturation decreases the incidence of blastocysts and increases the number of apoptotic nuclei and expression of apoptosis-associated genes in porcine embryos. Although apoptosis is an in vivo or in vitro physiological process that occurs during preimplantation embryonic development [46], compared with early cleaving, there is a higher incidence of apoptosis during late cleavage, and therefore the less functional embryo shows reduced developmental capacity [47]. There is also evidence in the literature that the expression of genes related to apoptosis, such as *Bcl-2* and *Bax*, is altered in embryonic culture [48]. In addition, expression of the *Bax* gene is increased in morphologically poor embryos compared with that in embryos with a good developmental ability [49]. Thus, these observations suggest that exposure to atrazine during oocyte maturation impairs the further developmental potential of porcine preimplantation embryos.

In conclusion, our results revealed negative effects of atrazine exposure on porcine oocyte maturation; this finding provides further evidence of the toxic effects of atrazine on reproductive systems.

## Supporting information

S1 File Raw data of Figs 1–7. (XLSX)
