# Correction: Toxic effects of atrazine on porcine oocytes and possible mechanisms of action

**DOI:** 10.1371/journal.pone.0362423

**Published:** 2026-11-10

**Authors:** Bao Yuan, Shuang Liang, Yong-Xun Jin, Ming-Jun Zhang, Jia-Bao Zhang, Nam-Hyung Kim

There is an error in Fig 1A in this article [1]: the Control panel was duplicated in the Atrazine (50 µM) panel. The correct Fig 1A is provided with this notice, and the image data provided in support of this figure are in S1 Data.

NHK and JBZ stated that the quantified results in Fig 1B are not affected by the above error.

The authors apologize for the error in the published article.

## Supporting information

S1 Data Original image data underlying Fig 1. (ZIP)

**Fig 1:**
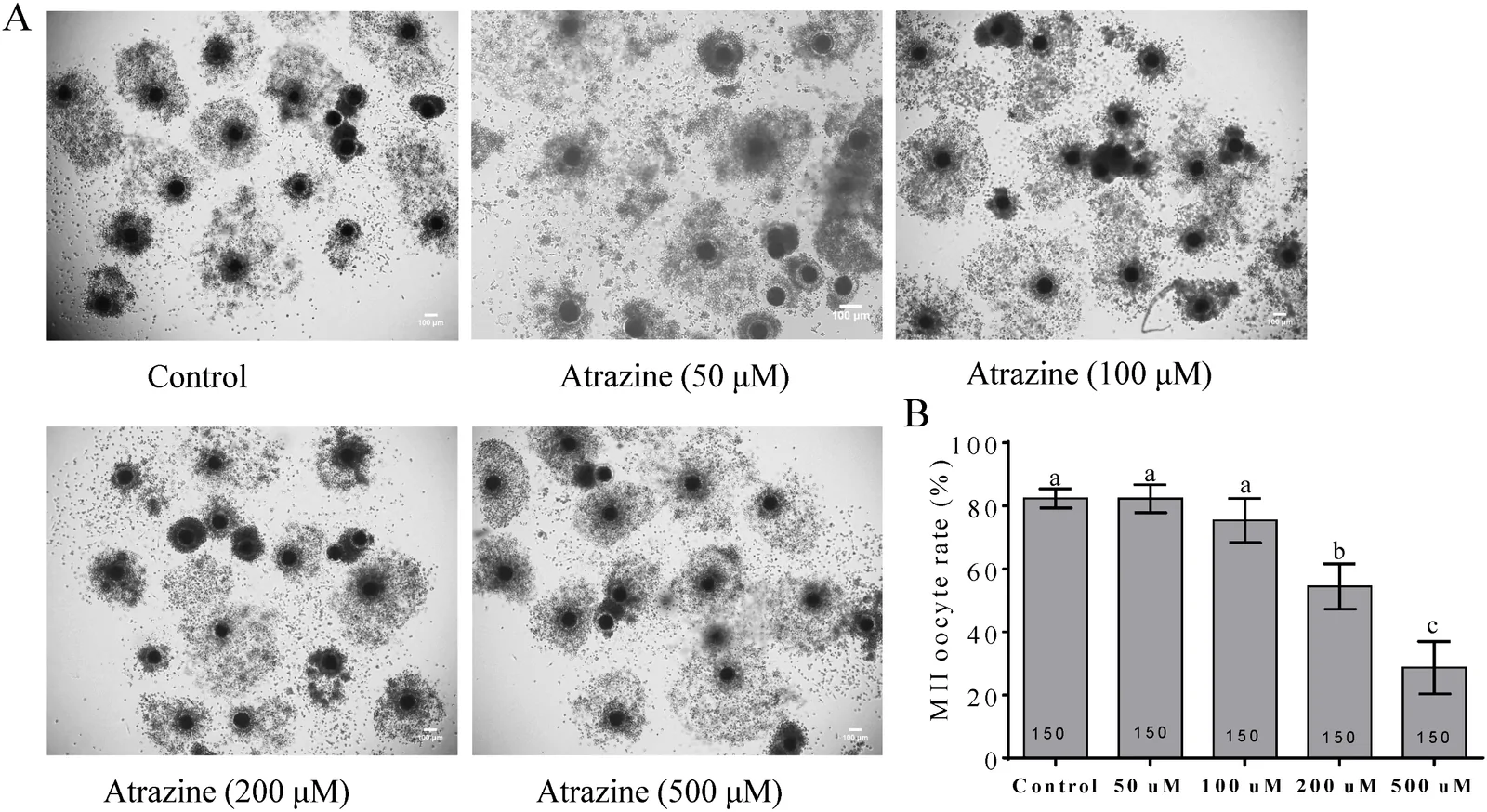
Effects of various concentrations of atrazine on IVM of porcine oocytes. (A) Expansion of the peripheral layers of cumulus cells. (B) The polar body extrusion rate was significantly reduced after atrazine treatment. Control: a normal IVM medium; 50–500 μM: different concentrations of atrazine. Differences between bars superscripted with different letters (a, b, or c within the same graph) are statistically significant (p < 0.05). Values are shown as mean ± standard deviation from three independent experiments. The number of oocytes observed in each experimental group is displayed in the bar.
