# Supplementary material for: Correction: Toxic effects of atrazine on porcine oocytes and possible mechanisms of action
Source: PLoS One. 2026 Nov 10;21(11):e0362423. doi: 10.1371/journal.pone.0362423 (PMC13750540; doi:10.1371/journal.pone.0362423)
Supplement: Supplementary file 1 [file pone.0362423.s001.zip › e0179861-Fig 1A.-raw data.pdf]

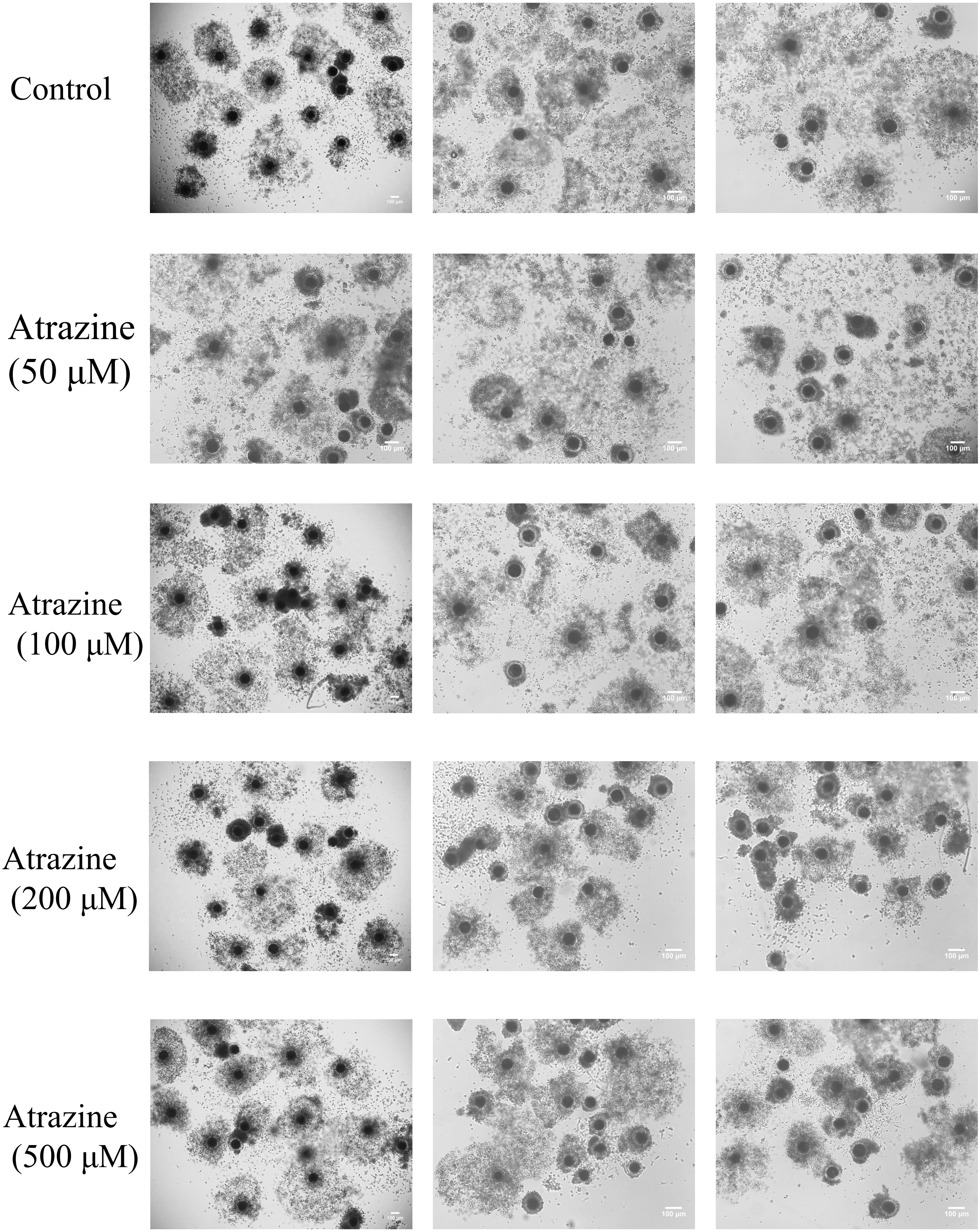

Control
Atrazine (50 μM)
Atrazine (100 μM)
Atrazine (200 μM)
Atrazine (500 μM)
